# Overcoming Shame to Vocalise During Childbirth: A Qualitative Interview Study

**DOI:** 10.1111/jan.70034

**Published:** 2025-06-26

**Authors:** Laura A. Zinsser, Nancy I. Stone

**Affiliations:** ^1^ Hannover Medical School, Midwifery Research and Education Unit Hannover Germany

**Keywords:** agency, childbirth, cultural norms, labour, midwifery, qualitative, shame, vocalising

## Abstract

**Aim:**

To explore women's experiences of vocalisation during childbirth and the interplay with their feelings of shame.

**Design:**

This interpretive, qualitative study is grounded in a social constructionist theory of gender, which provides a lens for examining the role of vocal toning in childbirth.

**Methods:**

Between November 2023 and March 2024, 18 women in Germany were interviewed postpartum either at home or virtually. Semi‐structured interviews were analysed using reflexive thematic analysis.

**Results:**

Two themes were generated: ‘the shame of being heard’ and ‘the value of my voice’. The findings demonstrate challenges women faced when vocalising during childbirth due to feelings of shame. Overcoming social norms were key; previous experience with vocalisation was an advantage. Vocal expression was also facilitated by a safe, supportive birth environment.

**Conclusion:**

Shame influences behaviour during childbirth. A key factor appears to be whether women feel safe and uninhibited in their vocal expression, which can be supported through empathic birth companions. Additionally, vocalising with others can contribute to normalising the experience and reducing feelings of shame.

**Implications for the Profession Care:**

The findings suggest that healthcare professionals can help women overcome shame to use vocalisation as a tool to navigate the challenges of childbirth.

**Reporting Method:**

Reporting adhered to the SRQR checklist.

**Patient and Public Involvement:**

This study did not include client or public involvement in its design, conduct, or reporting.

**Summary**

- What problem did the study address?
  ○ Cultural norms can inhibit vocalisation during childbirth.
- What were the main findings?
  ○ Women benefit from an environment where they feel empowered to use their voice without fear of judgment or shame.
- Where and on whom will the research have an impact?
  ○ This research can impact cultural and personal perspectives on vocal expression during childbirth, encouraging providers to create a supportive environment for self‐expression during childbirth.

## 1 Introduction

Shame is a fundamental and universally experienced affect, emerging when individuals perceive themselves as falling short of expectations or when they feel seen by others (Lewis 1995). Silvan Tomkins' (1987) foundational work on affect theory laid important groundwork for the study of shame, which is understood as both an internal emotion and a cultural construct. Shame can be triggered by internalised societal or gender constructions, or by events placing one outside of acceptable behavioural expectations (Lewis 1995). It is distinct from guilt, which is rooted in actions, whereby an individual experiences themselves as having done something bad. Conversely, shame centres on the self, evoking the thought, ‘I am not okay’, whereas guilt finds expression in ‘my actions are not okay’ (Tangney 2001; Sedighimornani 2018). Guilt often motivates reparative action, encouraging individuals to amend their wrongs, while shame is characterised by withdrawal, avoidance, and feelings of exposure and inadequacy (Nathanson 1992; Brown 2006). This distinction illustrates how shame targets first and foremost a person's identity, which can have an isolating and pervasive effect, subsequently impacting an individual's behaviour. Shame inhibits enjoyment and interest, is sensed as vulnerability towards that which is unfamiliar, such as an unknown person, and is an interruption to intimacy and closeness (Larsson 2024).

Physiological signs of shame include averted gaze, slumped posture, and a desire to shrink or disappear (Tangney 2001; Dolezal 2015). For this reason, it can be said that shame is profoundly embodied, manifesting physically in ways that reflect its intensity. These physical responses highlight shame's connection to the experience of being ‘seen’ by others, where the body itself becomes an object of scrutiny (Martin 2003). This transformation—from subject to object—can be triggered by the gaze of others or even from a so‐called internalised observer, deepening the sense of exposure and vulnerability (Bartky 1988). This embodied experience of shame is particularly significant for women, whose bodies are often culturally constructed as sites of regulation, judgement, and control (Tangney 2001; Martin 2003; Dolezal 2015).

Shame is shaped by societal and cultural norms, particularly in the context of gender. Women experience gendered shame, which is rooted in societal expectations that demand passivity, selflessness, and restraint (Bartky 1988). Sandra Bartky (1990) characterises this as a persistent emotional condition arising from patriarchal structures that regulate and discipline women's behaviour. This type of shame often requires women to navigate unattainable cultural ideals of femininity, which emphasise quietness, composure, and control even during moments of significant physical and emotional intensity, such as childbirth (Martin 2003). As a result, gendered expectations contribute to habitual shame, as women internalise these cultural demands and judge themselves against unrealistic standards (Brown 2006; Dolezal 2015).

## 2 Background

### 2.1 Shame and Childbirth

While childbirth is an intensely physical and expressive process and often reveals gendered dynamics, a sense of shame around experiencing the female reproductive cycle begins with most women at the commencement of menstruation, which is perceived to some degree as repugnant (Moloney 2010). It is often kept secret and is an aspect of comprehensive reproductive shame (McHugh 2020). Societal and cultural narratives surrounding childbirth perpetuate reproductive shame, framing women's experiences within strict norms, while media portrayals frequently sanitise or dramatise birth, obscuring the visceral realities of labour and reinforcing stigmas about women's bodily and vocal expressions. For example, in documentary‐style reality television, specific scenes and depictions of births are selected to create heightened emotional effects (Luce et al. 2016; Roberts and Benedictis 2019; Roosevelt et al. 2025). These portrayals entrench the expectation that women should be silent, composed, and restrained during childbirth, making shame a pervasive undercurrent in many birthing experiences.

Shame during childbirth is not only a reflection of external societal pressures but also an internalised force, shaped by what Karin Martin (2003, p.55) refers to as the ‘internalized technologies of gender’. Women often carry deeply ingrained standards for politeness, selflessness, and a desire to conform to societal expectations during childbirth, even in the face of profound physical pain and emotional challenges (Martin 2003; Roosevelt et al. 2025). This self‐disciplined behaviour frequently leads women to suppress their needs or self‐expression, reinforcing the cultural scripts that prioritise silence and control over autonomy and agency. Internalised discipline can also show up as reluctance or fear to be loud during labour. According to Penny Simkin (2011), healthcare providers can feel uncomfortable when labouring women's vocalisations during contractions are unusually loud, prompting them to offer pain relief medication. Karin Martin (2003) found in her study of women's experiences of their labouring body that birthing women sought to take care of others during childbirth, attempting to act selflessly to ensure that those present felt at ease.

### 2.2 Vocalising and Vocal Toning

Women^1^ often vocalise when they have contractions during childbirth. The sounds they produce range from breathing sounds, hums, moans, and vocal toning, to roars and primal screams (McKay and Roberts 1990; Baker and Kenner 1993; Pierce 1998; Danford and Mercein 2017; Zinsser et al. 2025). Vocalising is an umbrella term for all sounds made during childbirth. Vocal toning refers to long and full sounds, often vowels, that last the entire exhalation (Zinsser et al. 2025; Snow et al. 2018). In addition to this, the volume and intensity of vocalisations tends to increase as labour progresses (McKay and Roberts 1990; Baker and Kenner 1993; Valente et al. 2025). For many women, these sounds are unfamiliar and evoke a sense of alienation, causing them to withdraw (Danford and Mercein 2017). Moreover, vocal toning can sometimes resemble sounds associated with sexual arousal or expressions of emotion, making it a deeply personal and potentially vulnerable form of self‐expression (Zinsser et al. 2025). In some cases, this can contribute to feelings of discomfort or inhibition, as individuals may perceive this as an intimate disclosure, which they prefer to keep private (Anikin et al. 2018; Zinsser et al. 2025). Childbirth offers an opportunity to examine how women navigate and negotiate shame and self‐expression. This paper explores the interplay of shame and vocalisation during childbirth, particularly women's experience of the cultural discourse surrounding shame, their self‐expression, and their use of voice.

## 3 The Study

The aim of this study is to examine women's experiences of vocalisation during childbirth, with a particular focus on the interplay between vocal expression and feelings of shame. Specifically, it explores how cultural narratives, self‐expression, and voice shape the birthing experience, while also considering the conditions that support vocalisation—including self‐acceptance, bodily awareness, and the influence of supportive birth environments, as well as midwifery care.

## 4 Methodology

### 4.1 Design

A qualitative research design with an interpretative approach was employed to explore the experiences of women who vocalised during labour and birth. The authors of the study adhered to the Standards for Reporting Qualitative Research (SRQR) to ensure rigour and transparency (O'Brien et al. 2014).

### 4.2 Theoretical Framework

This study is informed by a social constructionist theory of gender, which posits that gender is not a biologically determined characteristic but rather a socially constructed aspect of identity that is continuously reinforced through interactions with others (West and Zimmerman 1987; Butler 1999). During labour and birth, women navigate cultural expectations that shape and impact their behaviour, physical expressions, and vocalisations (Luce et al. 2016; Danford et al. 2023; Roosevelt et al. 2025). From this perspective, gender is not a fixed or inherent trait but an ongoing performative act—one that is socially enacted, reiterated, and legitimised through routine practices and institutional norms (West and Zimmerman 1987).

### 4.3 Study Setting

In Germany, every pregnant woman is entitled to attend an antenatal course, which is financed by the statutory health insurance companies and includes up to 14 h of instruction. The content is tailored by the midwife providing the course (GKV 2025). In addition to information about the birth process, the puerperium, and breastfeeding, these courses often include relaxation and breathing exercises. Some midwives provide information or incorporate vocal toning exercises inspired by Frédérick Leboyer.

Women anticipating a physiological childbirth can freely choose their birth place in Germany. Options include obstetric‐led care in hospitals, midwife‐led care in maternity wards attached to an obstetric‐led labour ward, free‐standing birth centres, or home birth.

### 4.4 Participant Recruitment and Sampling

Eighteen women were recruited in the southwest of Germany at antenatal classes and postpartum via midwifery networks. In‐person recruitment efforts involved distributing study information via midwives, ensuring potential participants were informed about the study's objectives and data handling procedures. Interested individuals contacted the researcher via email or telephone. Women gave written consent at the interview. Recruitment continued until data saturation was reached, defined as the point at which no new themes emerged and key topics, such as vocalisation during labour, were consistently repeated across interviews. Saturation was determined to have occurred by the 17th interview, following established guidelines for assessing thematic saturation in qualitative research (Hennink and Kaiser 2022). Eligibility criteria included women aged 18 years or older, who had given birth to a healthy baby within the past 6 weeks, had experienced spontaneous vaginal birth without pharmacological or medical interventions (e.g., epidural analgesia or oxytocin augmentation), and had vocalised at one point during labour or birth. Women who had undergone planned caesarean sections or received pharmacological pain relief were excluded from the study. Eligibility criteria were assessed prior to each interview, therefore none of the participants were deemed ineligible.

### 4.5 Data Collection

Semi‐structured interviews were conducted between November 2023 and March 2024, either in participants' homes or virtually via MS Teams, depending on participant preference. Interviews were carried out in environments conducive to comfort and open discussion, including living rooms, bedrooms, and kitchens. Participants were encouraged to freely express their experiences, with interview prompts designed to elicit in‐depth responses, such as ‘Can you describe your experience with vocalising during labour?’ Interviews lasted between 30 and 79 min and were audio‐recorded for transcription and analysis. The newborns were present during all interviews, and participants were given the flexibility to pause for breastfeeding or other needs.

### 4.6 Data Analysis

A reflexive thematic analysis approach, as outlined by Braun and Clarke (2022), was utilised to analyse the data. The analytical process followed six interconnected phases: (1) familiarisation with the data, during which each interview transcription was read multiple times to get immersed in the content; (2) generation of initial codes, where a line‐by‐line, inductive analysis was conducted and iteratively refined throughout the coding process; (3) theme development, wherein patterns were identified and organised within the codes that provided specific insights into the research question (4) theme review, involving the critical evaluation, modification, and further development of preliminary themes; (5) theme definition and naming, in which the essence of each theme was clearly articulated and labelled; and (6) writing up and reporting findings, where the final themes were woven into a coherent analytic narrative presented in this paper. Initially, two interviews were independently coded by both authors to ensure consistency and to establish a shared understanding of the data. The first author then coded the remaining transcripts. Regular discussions were held to refine the coding framework. Special attention was given to the spontaneous vocal reproductions by participants during interviews, incorporating both descriptions and actual sound expressions into the analysis (Zinsser et al. 2025).

### 4.7 Researcher Reflexivity

The research team comprised two postdoctoral researchers with backgrounds in midwifery. L.A.Z. is a midwife and academic researcher in the field of midwifery. She is an expert in physiology of childbirth based on her working experience in midwife‐led settings. For her, vocalisation during labour and birth is an integral part of giving birth. During the interviews, she tried to create a safe space and have an open and accepting attitude.

N.I.S. is a midwife and academic researcher in the field of midwifery. She has experience as a midwife in hospital labour wards and in a free‐standing birth centre and is currently working in a hospital labour ward, where vocalising during labour is encouraged. She has found that both women's and midwives' acceptance of vocalisation during childbirth varies depending on the healthcare practitioner present at birth, the woman, and the institutional environment.

In order to have a deeper experience of the subject matter, L.A.Z. and N.I.S. met to discuss and reflect on sound and vocalisations during childbirth. Reflection broadened their understanding and openness to different experiences, in particular to the experience of shame and sound production. An open attitude and an awareness of their subjectivity were also mutually reflected upon when analysing, discussing, and debating the findings.

### 4.8 Ethics Statement

The ethics committee has approved this study in September 2023 (Nr. 11060_BO_K_2023) at Hannover Medical School. Participants were provided with written information and informed that they can choose not to respond to any questions in the interview or discontinue the interview at any time.

### 4.9 Rigour and Trustworthiness

To ensure the study's rigour, several strategies were employed, drawing on the criteria of credibility, transferability, dependability, and confirmability as outlined by Lincoln and Guba (1985). Credibility was reinforced through engagement with participants until data saturation was reached, as well as through reflexive practices. Transferability was addressed by providing detailed descriptions and participant quotes, enabling readers to assess applicability to their clinical contexts. Dependability was ensured through team‐based coding and ongoing discussions during analysis, while confirmability was ensured through regular documentation and audit trails that were maintained throughout the study. By employing these methodological strategies, the study provides a robust exploration of the ways in which shame manifests in the vocal expressions of women during childbirth, offering valuable insights into their experiences within cultural and societal frameworks.

## 5 Findings

The two themes (Table 1) that were generated, ‘the shame of being heard’ and ‘the value of my voice’, exhibit the interplay of the research participants' internal experiences, their engagement with their environment and those who accompanied them, as well as the steps they took to overcome shame. Participants' demographics are presented in Table 2.

**TABLE 1:** Themes and subthemes of overcoming shame to vocalise during childbirth.

Theme 1: the shame of being heard	Theme 2: the value of my voice
Showing my true self	Inner readiness to accept my voice
Perceiving myself outside social norms	Connecting with my body to free my voice
	An environment that encourages self‐expression

**TABLE 2:** Demographics of the participants.

Participant	Age	Gravida/Para	Place of birth
Fenja	31	1/1	Hospital
Franziska	33	1/1	Hospital
Maria	34	1/1	Home
Vanessa	35	1/1	Hospital
Johanna	33	2/2	Home
Cassandra	36	2/2	Free‐standing Birth centre
Natalie	31	2/2	Home
Anna‐Maria	27	2/2	Home
Felizitas	28	2/2	Home
Lisa	35	2/2	Hospital
Melanie	39	2/2	Home
Christina	40	2/2	Hospital
Veronika	36	2/2	Hospital
Ruth	38	4/2	Home
Linda	38	3/3	Home
Helene	38	3/3	Home
Judith	41	4/4	Home
Tamara	44	5/4	Home

### 5.1 Theme 1: The Shame of Being Heard

The first theme, ‘the shame of being heard’, generated two subthemes: ‘showing my true self’ and ‘perceiving myself outside social norms’. The subtheme **‘showing my true self’** captures the realisation that their vocalisations were like an exhibition of their innermost selves. The sounds they made during labour could not be confined to the immediate space where they were giving birth. Inevitably, these sounds could be heard in the surrounding areas, reaching people who were not directly involved in the birth. This realisation made it challenging for some women to initially vocalise freely. They equated the act of vocalising during labour with perhaps exposing their true self to people they did not want to include in their experience and thus felt reluctant to make sounds.

Many of the study participants lived in apartment buildings. Natalie, who was labouring at home, explained that she had no control over whether others could hear the sounds she made during labour.

I thought that the other people in my building, might be awakened. During the birth of [my first child], the neighbours woke up. And I wondered what they might be thinking and so on—I don't know. [I was] ashamed of the intimacy and that maybe someone else would [hear] me. (Natalie, Home)

Vocalising in front of others was perceived as an intimate act and was particularly challenging. Ruth, who was pregnant with her second child, described feeling self‐conscious when she practiced vocal toning during a birth preparation class because of the intimate nature of making labouring sounds in front of others. She explained:

In a birth preparation class, it's difficult because it's so intimate and so many other people are there. It is just like singing in front of others, which is super intimate. I think there's this threshold of inhibition that can influence you if you don't see it as something natural, that people have a difficulty with this. (Ruth, Home)

Some participants felt that they were being observed during labour and had difficulty with this. When they vocalised, this feeling intensified, since they knew that they could be heard by others. They felt vulnerable because they perceived themselves as being seen. Felizitas explained her challenge to deal with having the full attention of others and to continue to vocalise during contractions in spite of this. She said:

What I felt was, because I am the only one who was loud and making strange sounds sometimes, that it was a bit like, when you stand on the stage and sing, and everyone hears you. It was a bit like that. You are anyway the centre of attention, and then you're so loud. […] [At birth] you are not alone; others are present who are so focussed on you and are listening really attentively to you, that it really makes a difference with your ability to make any sounds at all. (Felizitas, Home)

In this subtheme, it was shown that women experienced shame when they perceived that they were the centre of attention and being watched or heard, making it difficult for them to vocalise. Many expressed that they could not fully escape feelings of shame, particularly when making sounds such as vocal toning or moaning. For these women, labour and birth were deeply intimate experiences, and making sounds during the process felt like sharing that intimacy with others.

The subtheme **‘perceiving myself outside social norms’** explores the tension women experienced between their urge to conform to societal norms and their need to vocalise during labour. For many participants, vocalising loudly felt shameful, as it conflicted with ingrained social expectations about how women should behave. These norms often reflected broader cultural narratives, including portrayals of childbirth in movies or media that depict women screaming, which some participants found alienating and unrelatable.

Many women expressed feeling uncertain in general about how they should behave during labour and birth. Christina, who watched labouring women on social media during her pregnancy, asked herself if she would be able to make sounds during labour, specifically moaning sounds. She shared:

When I watched it [vocalising during labour] on Instagram, it was really alienating. It wasn't like I said: it's not my thing. […] And I think that what was so alienating was that I couldn't grasp it. And then I thought, is it all right, is it okay if I moan? Is it, or isn't it? Although I know that's really stupid; everyone can experience their birth how she thinks is best and everyone is allowed to do what makes them feel good. (Christina, Hospital)

A typical question that some of the participants expressed asking themselves during labour was if they were disturbing other people with the sounds they were making. Cassandra, who was giving birth to her second child, said:

I had a lot of things going on [in my mind], like if I'm allowed. Is it uncomfortable for anyone here? Is it unpleasant for me? So many norms that have to be broken through. (Cassandra, FSBC)

Many participants expressed that the volume of their vocalisations felt problematic. Being loud often conflicted with their sense of how they were expected to behave in the presence of others. Christina specifically described the social norms that shaped her perceptions of being loud during labour and birth, linking these norms to broader attitudes towards other types of vocalisations. For her, the message, such as the idea that loudness, especially from children, is unwelcome, had an effect on her own self‐expression. She shared:

Yes, and I think sometimes it's like this, this external influence, what society kind of mirrors back to you, yes, where it's never really welcome or, well, when children scream, it's unpleasant, and then you also kind of suppress it, unconsciously, I believe. Yes, because you don't really have any access to it, I would say. (Christina, Hospital)

Building on this, she highlighted a double standard in how loudness is perceived in men versus women. She noted:

When I'm at the gym—I worked there for a while—and the men, when they lifted weights, they moaned and screamed and that was okay. But if a woman had stood there and screamed, people would have thought: Hey! Seriously, what's wrong with her? (Christina, Hospital)

Anna‐Maria was more comfortable being alone during labour because she felt ashamed of the volume of her vocalisations when others were in the room. She said:

So, somehow, I always wanted to be alone, and, I don't know if that was maybe because for me—because I am probably someone who is usually not loud, and I have some issues with noises (laughs). And this was probably why I started relatively late during my first birth to vocalise. (Anna‐Maria, Home)

Vocalising and vocal toning were both experienced by several participants as negative. Some felt that being loud during labour was at odds with societal values that discourage women from making noise or audibly expressing pain. Others noted that the double standards in how men's and women's loudness is perceived can lead to judgement or discomfort. They inferred that vocal toning is not widely accepted in society and that it conflicted with their personal values and self‐image.

### 5.2 Theme 2: The Value of My Voice

The second theme ‘the value of my voice’ shows how women embraced the normality of vocal self‐expression and overcame shame during childbirth. This theme has three subthemes: ‘inner readiness to accept my voice’, ‘connecting with my body to free my voice’ and ‘an environment that encourages self‐expression’.

The subtheme **‘inner readiness to accept my voice’**, illustrates how women discovered their voice during labour and birth. Women found ways to overcome shame through inner acceptance, which led to a willingness to vocalise. To overcome shame, some women reported needing to cross an internal threshold before they could produce sounds. This involved overcoming multiple barriers to self‐expression, particularly the challenge of breaking away from social norms. Linda, who vocalised during the birth of her third child, described the need to summon courage to express herself. She also expressed feeling uncertain about whether vocalising was acceptable. She said:

You have to have courage. With the other [births], I didn't really have so much courage and I thought, okay, I don't want to seem odd. (Linda, Home)

Participants who had vocalised in a previous birth recognised the benefits of vocalising during contractions, having internalised it as a resource to manage their pain. This prior experience gave them the confidence to use their voice again. Anna‐Maria, whose previous birth was imbued with her vocalisations, felt comfortable to use her voice again. She said:

[With this birth] I knew that it [vocal toning] is good for me and that's why I started early on with it. (Anna‐Maria, Home)

Some women who had never given birth before cited a lack of information about the positive effects of vocal toning as a barrier to actively engage in vocal toning. Several participants highlighted the importance of understanding vocal toning and its role in managing contractions as a way to overcome inner resistance and barriers. Franziska recalled trying to suppress the sounds her body instinctively wanted to make until she could no longer hold them back. She later wished she had known earlier the relief vocalising could provide. She said:

Then I would have known that, if I vocalise now, it might bring me relief. And then I might have consciously started much earlier to vocalise, instead of waiting until it was no longer possible to hold back, and my body started doing it on its own. I might have just started earlier and not suppressed anything. I think I would have found it very important for this to be properly discussed or addressed. You know, you kind of see it in some films or something. I know that sounds are made [during labour and birth], but that's all completely removed from reality. (Franziska, Hospital)

For some participants, vocalising during childbirth required an internal shift in mind‐set—a conscious decision to accept their own voice as a supportive tool. The readiness to vocalise was not just about external exposure but also about developing a sense of confidence in expressing sound. Christina, who gave birth to her first child, reflected on how her initial reluctance stemmed from feeling uncertainty. She shared:

But what's like, yeah, you hear about birth, yeah, the screaming, sure, but then I think (.) I don't really want to do that either. But I think if it [vocalising] wasn't so strange and I had already been more familiar with it and told myself‐ *Okay, I'll try it* ‐ and it's normal and just supportive because of this or that, then I think it wouldn't have felt so strange to me. (Christina, Hospital)

For some participants, the enjoyment of singing created an openness to vocalise during labour. A few women reported that they already liked to sing, which made them more willing to use their voice during contractions. Through singing, they had become accustomed to the familiarity of their voice. Maria shared how their love of singing influenced their approach to vocalising during labour:

No, I didn't decide in advance to do it [vocalise]. What I thought is that it probably would happen because I really like to sing, for example. That I definitely vocal ton. (Maria, Home)

Experiencing vocalisation without shame required inner acceptance and a willingness to produce sound. This acceptance was shaped by previous experiences of vocalising during birth, knowledge of its benefits for managing contractions, and an existing familiarity with using their voice, such as through singing before pregnancy. These factors motivated participants to use their voice during labour and birth, helping them to overcome internal resistance, to surrender to the process, and to express themselves freely.

The subtheme **‘connecting with my body to free my voice’** describes how women shifted their awareness into their bodies, enabling them to vocalise freely. A key factor in feeling at ease with vocal toning—without experiencing shame—was remaining present in their physical sensations rather than engaging in intellectual self‐monitoring. Many women described this as an inner willingness to release resistance and fully engage with the process. For Fenja, who gave birth to her first child, vocalising became a symbol of her complete dedication to the birth process.

Well, for me vocalising was a bit like surrendering. (Fenja, Hospital)

A few of the women practiced vocal toning during pregnancy on their own to prepare for their birth. Linda emphasised the importance to actively engage with her inner willingness to let go. During her third pregnancy she chose to prepare herself mentally so that she would be ready to vocalise and allow the sounds to emerge freely during labour.

Opening up and letting go [cognitively]. I think that's the step to take before the birth begins—asking yourself, well what does it actually take in this moment to be able to do it [to vocalise]. (Linda, Home)

Other women reported that it was important for them to be attuned to their feelings. They did this by connecting with their body and performing self‐assessment. Asking themselves the question: ‘How do I feel?’ helped participants recognise when vocal toning felt coherent and beneficial. Lisa described how she connected to her physical sensations to find what was right for her. She discovered that vocal toning provided a sense of alignment and coherence in that moment:

Actually, already at the beginning, when the contractions weren't yet so strong, I could manage with quiet vocal toning sounds. Also, it was simply a natural reaction to [me asking myself] what would be good for me at the moment. (Lisa, Hospital)

The feeling of coherence primarily guided participants' awareness of their bodies in relation to vocal toning. Cassandra experienced a deep sense of coherence and naturalness in her vocalisations:

It was definitely–like the voice of birth said to me that I should do it [vocalise]. And more like not resisting something that is naturally unfolding. I had a very strong feeling of naturalness when making vocal toning sounds. That it feels right and normal and that this is actually how it should go or could go. That it is a relief and is not forced at all. (Cassandra, FSBC)

During labour and birth, participants described reaching a point where they allowed their body to take over the work of giving birth and stopped assessing the sounds they were making. When they felt their bodies take over, they were no longer in their intellect and were freely able to make sounds. Franziska, reflecting on the birth of her first child, shared how vocal toning emerged instinctively when labour became intense.

It was uncomfortable for me at the beginning, and, because of that, I tried to suppress it. And then, at some point, I didn't care anymore. You just can't manage well anymore at some point, especially when the pain gets really extreme—then I simply had let it out. (Franziska, Hosptia)

Making a conscious decision to surrender to the birth process helped women to engage with vocalising. Some made this decision during pregnancy, while others had this experience only after labour had begun. Participants also found it helpful to focus their awareness on their body and be attuned to their sensations. Being present in their body rather than in their intellect during labour and birth helped participants to use their voice.

The subtheme **‘an environment that encourages self‐expression’**, highlights the external conditions that supported women to free their voice during labour. Nearly all the women described the importance of feeling safe and secure in their birthing environment as a requirement for vocal toning. A welcoming attitude of those accompanying them during labour and birth towards all types of vocalisations was essential. Some women described how midwives vocalised with them, which helped normalise the experience and made it easier to let go of shame. Many emphasised the importance of feeling at safe so that they could fully be themselves. Franziska shared that giving birth in a hospital reassured her, as she believed the staff understood vocalisation as an inherent part of labour, allowing her to vocal tone freely.

I thought it was really—when I think back on the birth, it was absolutely wonderful. I have to say, I didn't feel uncomfortable at all or anything. (‐) And I knew I could vocalise and do what I needed. Because they [the staff in the hospital] experience births every day. (‐) And everyone—or every woman—does her own thing. (Franziska, Hospital)

A sense of comfort was especially meaningful for the interviewees enabling them to express themselves freely in their chosen place of birth without feeling the need to conform to expectations. Linda, who had a home birth, perceived that trusting the people around her was essential in helping her open up and vocal tone. She explained the conditions she needed to make vocal toning possible:

Trust in myself, truly, that it's okay the way I'm doing it in that moment. Trust also in the people accompanying me—my partner and midwife, truly. This willingness to open up, I think that is very important. And simply the feeling as a woman that the midwife accompanying me in that moment values me and that I can and am allowed to be just as I am. This acceptance or complete acknowledgment that I don't have to pretend to be something I'm not, you know? And very important for me was being at home in my own environment, knowing that there aren't strangers behind the next door who could hear me or might come in. (Linda, Home)

For many women, supportive midwives played a key role in encouraging vocalisation. When women perceived signals from the midwife that vocalising was acceptable or even desirable, it helped them feel more at ease. Veronika described how her interaction with her midwife reinforced this acceptance. She shared:

But I did feel safe at the hospital, so I think I was able to let it out somehow. And it was also very clear because I had spoken with the midwife beforehand, while the breaks between contractions were still long enough, that she would always stop talking when she noticed a contraction was coming, and then, you know, like that. So, it was a clear signal from her, like, ‐ *Okay, now your contraction is starting, focus on yourself, uh, focus on yourself* ‐. (Veronika, Hospital)

Some participants wished they had received explicit verbal reassurance from their midwife that vocalising was not only acceptable but welcomed. Felizitas, who gave birth at home, reflected on how such verbal support from her midwives may have minimised her sense of shame while vocalising:

…that she [the midwife] would have said to me something like ‐ *so now you're in a safe space here* ‐. If she had really said that with words, I would have known that it's a safe space. But if she would have said that it's a safe space and I can vocalise as loud as I want and make all the noises—I think that would have helped me to have felt less ashamed. (Felizitas, Home)

Beyond midwifery support, it was important for the interviewees that their birth companions accept their vocalisations. One woman noted that, during her second pregnancy, she deliberately informed her partner in advance of her intention to vocalise during labour:

Cassandra: My husband knew already from my previous birth [that I vocalise] and I told him that it would be similar this time.

Interviewer: Was it important for you to tell him this in advance?

Cassandra: Yes, it was important to me because embarrassment and shame go along with vocalising. (Cassandra, FSBC)

For the interviewees, the strongest reinforcement for vocalising during labour was to vocalise together with others. This not only normalised their vocal expressions, but also fostered a sense of connectedness, where shame no longer played a role. Linda described her experience of vocalising with her midwife, after being asked what it was like for her to vocal tone with others:

I liked it. For me/because I was already so sure of myself and in my trance state, I found it good. But I think for women who don't dare to do it, it's really supportive. It creates this sense of community, you know? It takes away this special, this, well—*I'm vocalising alone*—it removes the stigma, so to speak, and you get the feeling that it's something completely normal, that we're all vocalising a bit together now. (Linda, Home)

A supportive birthing environment was essential in empowering women to vocalise during labour. The openness and support of midwives and birth companions played a pivotal role in fostering this atmosphere. Participants valued when vocalisation was explicitly acknowledged and encouraged as a beneficial and integral aspect of labour. Notably, vocalising alongside others proved to be a powerful motivator, reinforcing the perception of vocalisation as an integral and normal element of childbirth, thereby reducing feelings of shame.

## 6 Discussion

In this paper we explored women's experiences of vocalisation during childbirth and the interplay with their feelings of shame. Shame triggered an internal resistance to vocal expression since they were uncertain if it was acceptable. Shame was particularly pronounced when women felt observed by those present at their birth. They found it challenging to be the centre of attention, which resulted in discomfort and a sense of being judged. When they perceived their behaviour as deviating from social and gender norms, they described encountering an internal threshold they had to overcome to fully use their voice (Sedighimornani 2018). A key factor in this was the uneasiness they felt being loud—especially as women (West and Zimmerman 1987). They worried about disturbing others, as dominant cultural expectations of women in western societies associate femininity with being nice, kind, polite, and selfless (Martin 2003; Deutsch 2007). When they were loud, they were not conforming to these norms and, therefore, were not ‘doing gender’ in the way that is socially expected of women (West and Zimmerman 1987). Being loud also conflicted with dominant ideals of femininity, which regard such behaviour as disruptive, rude, or even inappropriate (Butler 1999; Martin 2003; Roosevelt et al. 2025). Non‐conforming behaviour—particularly when it challenges societal expectations of women—can create a sense of self‐disruption, making individuals feel as though they are jeopardising their self‐image (Ferguson et al. 2000; Martin 2003). However, if gender is understood as a construct, as argued by Deutsch (2007), then it can also be deconstructed. In this study, women challenged gendered expectations around vocalisation through self‐reflection and the supportive presence of midwives and birth companions. By drawing on internal resources and engaging in affirming interactions, they were able to alleviate feelings of shame and resistance. At this point, it should also be noted that there were participants who only began to vocalise when the contractions were so intense that it was no longer possible for them to suppress their voice. Nevertheless, the findings indicate that gender‐specific shame is a complex yet modifiable experience, mitigated through both personal agency and external support. Through this process, women transcended shame and experienced greater freedom during childbirth.

### 6.1 Shame, Self‐Exposure, and the Challenge to Allow Authentic Vocal Expression

For many women in this study, vocalising externalised their internal emotional states and was an intimate experience through which emotions became audible to others, echoing Johan Sundberg's (1982) essay that vocal expression communicates emotional states. While gendered shame shaped the experiences of the women in this study, they also faced the broader challenge of being seen in their most vulnerable and authentic self‐expression. This confrontation with exposure and self‐revelation is not exclusive to women but reflects a fundamental human experience, as shame can arise when individuals feel scrutinised or exposed, regardless of gender (Tomkins 1987; Lewis 1995). Revealing one's innermost emotions can lead to embarrassment, prompting efforts to restore one's social image or standing in the eyes of others (Keltner et al. 2022). Shame is closely tied to the experience of belonging, as it involves the fear of disconnection and rejection (Brown 2006). This aligns with the broader assumption that individuals seek validation and inclusion within their social environments (Baumeister and Leary 1995).

Additionally, a perceived loss of control over vocal expressions can intensify feelings of shame (Lietzmann 2003). Anja Lietzmann (2003) suggests that some individuals conceptualise their body as an object, treating it as an instrument to be used and controlled. Individuals who treat the body as an object can experience that their uncontrolled physical and vocal expression is perceived as shameful (Lietzmann 2003). When individuals feel unable to regulate their bodily and vocal responses, this can contribute to a sense of disorientation regarding their own identity, triggering shame through the (un)conscious question: *Am I acceptable as I am?*—a concern raised by several participants in this study (Lietzmann 2003; Sedighimornani 2018; Roosevelt et al. 2025). When bodily or vocal expressions deviate from this perceived control, they may be experienced as shame‐inducing. In this sense, shame disrupts a person's sense of agency and self‐regulation (Lietzmann 2003). Lastly, our findings support the understanding of shame as a relational, interpersonal, and socially constructed experience (West and Zimmerman 1987), shaped by interactions with others rather than an inherent or solely internalised phenomenon.

### 6.2 Safe Space for Uninhibited Expression

Some women actively sought to maintain or restore their sense of belonging (Keltner et al. 2022). They did so by informing their partner during pregnancy about their intention to vocalise; choosing a supportive midwife who respected their wishes; and/or selecting a birth setting where they felt safe (Carlsson et al. 2020; Almorbaty et al. 2023). For some, the awareness that their vocalisations would carry beyond the walls of their birth room created a sense of inhibition. The presence of others, or the possibility to be heard, led some women to consider suppressing their vocal expressions. These instances might be read as spatial concerns, yet our findings suggest that what was at stake was not space itself, but the quality of relational safety within it. Our findings suggest that feeling safe and being able to express oneself during labour requires relationships built on trust and a fulfilled sense of belonging (Brown 2006; Keltner et al. 2022). Shame relates primarily to connection and the experience of feeling connected (Brown 2006). From our perspective, this sense of connection transcends the spatial context—whether public or private.

Women reported that vocal toning together with another person provided greater reassurance than receiving explicit verbal permission to vocal tone during labour. Shared vocal toning reduced feelings of isolation and fostered a sense of human connection, thereby normalising the experience (Miller and Stiver 1997; Mathias et al. 2021). This facilitated acceptance of one's own vocal expressions by transforming potential embarrassment or shame into feelings of connectedness and support. The act of vocalising together can be an empowering strategy for midwives to support their clients in finding and confidently using their voices. These types of shared experiences shift attention away from societal and cultural expectations towards affirming personal values and meaningful self‐expression (Brown 2006). Our findings indicate that participants, when they did experience shame, could readily overcome it when they felt a sense of connection to their midwife and partner (Brown 2006; Keltner et al. 2022). A compassionate approach by midwives, characterised by empathy, acknowledgement of emotions, and responsiveness to women's needs, was key to reducing uncertainty, fear, and internal barriers, thus enhancing connection and positively influencing the birth experience (Wiseman 1996; Rosenberg 2003; Moloney and Gair 2015; Krausé et al. 2020; Roosevelt et al. 2025).

### 6.3 Strengths and Limitations of the Work

One limitation of this study is that nearly all participants grew up in Germany, which could influence their attitudes towards the use of soun2ds during labour and birth. Cultural and linguistic factors may have shaped both their perceptions of vocalisation during labour and the types of vocal expressions they used. Additionally, participants were purposively recruited, introducing a potential risk of self‐selection bias. Only women who had vocalised during childbirth were included in this phase of the study; therefore, the findings may not reflect the broader spectrum of birthing experiences, particularly those of individuals who did not vocalise. Participants had varying levels of prior experience with vocalisation—some were familiar with it through yoga, social media (e.g., Instagram), or antenatal classes. Others were not previously aware of vocalisation or did not consciously vocalise during birth, and only reflected on the experience retrospectively, prompted by the midwife who told them about the possibility to participate in the study. In our sample, just one woman reported that she was unable to use her voice during her previous births due to shame and that during her third birth, she explicitly confronted her shame in order to be able to vocalise. This study focused on vocalisation as a way of coping with childbirth. No questions were asked specifically about shame. Given that the aim was to explore the experience of vocalising in childbirth rather than to investigate predetermined topics, this approach was deemed appropriate to the research aim. Hence, not all participants talked about shame in their interviews. Those who did contributed rich and detailed insights that enabled a deeper understanding of how shame may be associated with vocal expression in childbirth. Moreover, the participants' openness in sharing their experiences added considerable depth to the data and can be viewed as a strength of the research.

### 6.4 Recommendations for Further Research

We recommend that future research explore vocalisation during childbirth across a broader range of cultural contexts. Given that nearly all participants in this study had a Western cultural background, there is a need to understand how cultural norms and expectations shape vocal expression during labour in diverse settings. Further research should also focus on developing and evaluating strategies to support women in overcoming both gendered shame and voice‐related shame during childbirth. This could inform the design of antenatal education and midwifery care practices that foster more inclusive, respectful, and empowering birth experiences. Finally, it may be valuable to explore how midwives and birth attendants perceive and respond to women's vocal expressions during labour. Understanding provider attitudes could support more nuanced training approaches that help caregivers recognise vocalisation as a meaningful and potentially beneficial part of the birth process.

### 6.5 Implications for Policy and Practice

To help reduce feelings of shame during labour, healthcare professionals should introduce the topic of vocalisation during pregnancy, presenting it as a normal and potentially beneficial aspect of the birthing process. This includes openly discussing how vocal sounds can support women during labour and birth. Care providers are encouraged to reflect on and cultivate an open, non‐judgmental attitude towards vocal expression to foster an atmosphere where women feel acknowledged and supported. For women who struggle with shame, practices such as singing in preparation for birth, alongside conscious reflection on internalised social norms, may offer valuable support. Midwives should clearly communicate—both antenatally and during birth—that all forms of vocal expression, including loud vocalisation, are welcome. Joint vocalisation between the midwife and the birthing woman emerged as a powerful source of reassurance and permission. Offering vocal toning together in an empathic manner could be a meaningful way to normalise vocal expression and support women during labour.

## 7 Conclusion

This study set out to explore women's experiences of vocalisation during childbirth. Within this paper, the focus was on how vocal expression intersects with experiences of shame, particularly in relation to cultural norms, self‐expression, and the use of voice. The findings illustrate how internalised expectations and social scrutiny can inhibit vocalisation, while self‐acceptance, bodily awareness, and supportive environments—especially those fostered by midwives and companions—can help women overcome shame. Vocalising together emerged as a particularly powerful way to normalise expression and strengthen a sense of connection. Understanding these dynamics can contribute to more empathic, inclusive birth care.

## Author Contributions

**Laura A. Zinsser:** conceptualisation, methodology, validation, formal analysis, investigation, writing – original draft, writing – review and editing, project administration, funding acquisition. **Nancy I. Stone:** writing – original draft, intellectual input, writing review and editing.

## Conflicts of Interest

The authors declare no conflicts of interest.

## Data Availability

Data available on request from the authors.

## Endnotes

^1^
We acknowledge the diversity of gender identifications. We decided to use the term women in this manuscript, as the participants identified themselves as women.

## References

[jan70034-bib-0001] Almorbaty, H. , L. Ebert , E. Dowse , and S. W. Chan . 2023. “An Integrative Review of Supportive Relationships Between Child‐Bearing Women and Midwives.” Nursing Open 10, no. 3: 1327–1339. 10.1002/nop2.1447. 36349710 PMC9912441

[jan70034-bib-0002] Anikin, A. , R. Bååth , and T. Persson . 2018. “Human Non‐Linguistic Vocal Repertoire: Call Types and Their Meaning.” Journal of Nonverbal Behavior 42: 53–80. 10.1007/s10919-017-0267-y. 29497221 PMC5816134

[jan70034-bib-0003] Baker, A. , and A. N. Kenner . 1993. “Communication of Pain: Vocalization as an Indicator of the Stage of Labour.” Australian and New Zealand Journal of Obstetrics and Gynaecology 33, no. 4: 384–385. 8179547 10.1111/j.1479-828x.1993.tb02115.x

[jan70034-bib-0004] Bartky, S. L. 1988. “Foucault, Femininity, and the Modernaization of Patriarchal Power.” In Feminism & Foucault: Reflections on Resistance, edited by I. Diamond and L. Quinby , 25–45. Northeastern University Press.

[jan70034-bib-0005] Bartky, S. L. 1990. Femininity and Domination: Studies in the Phenomenology of Oppression. Routledge.

[jan70034-bib-0006] Baumeister, R. F. , and M. R. Leary . 1995. “The Need to Belong: Desire for Interpersonal Attachments as a Fundamental Human Motivation.” Psychological Bulletin 117, no. 3: 497–529. 10.1037/0033-2909.117.3.497. 7777651

[jan70034-bib-0007] Braun, V. , and V. Clarke . 2022. Thematic Analysis: A Pracitical Guide. SAGE Publications.

[jan70034-bib-0008] Brown, B. 2006. “Shame Resilience Theory: A Grounded Theory Study on Women and Shame.” Families in Society 87, no. 1: 43–52. 10.1606/1044-3894.3483.

[jan70034-bib-0009] Butler, J. 1999. Gender Trouble: Feministm and the Subversion of Identifty. 10th ed. Routledge. 10.4324/9780203902752.

[jan70034-bib-0010] Carlsson, I. M. , I. Larsson , and H. Jormfeldt . 2020. “Place and Space in Relation to Childbirth: A Critical Interpretive Synthesis.” International Journal of Qualitative Studies on Health and Well‐Being 15, no. sup1: 1667143. 10.1080/17482631.2019.1667143. 33103631 PMC7594831

[jan70034-bib-0011] Danford, K. , and J. Mercein . 2017. “The Birth Process and Voice Training: The Glorious Chorus.” VRS 12, no. 1: 35–48. 10.1080/23268263.2018.1417097.

[jan70034-bib-0012] Danford, L. , A. Roosevelt Vroom , L. Harris , and R. Zielinski . 2023. “Impolite Birth: Theatre Voice Training and the Experience of Childbirth.” Voice and Speech Review 17, no. 2: 167–181. 10.1080/23268263.2022.2137970.

[jan70034-bib-0013] Deutsch, F. M. 2007. “Undoing Gender.” Gender & Society 21, no. 1: 106–127. 10.1177/0891243206293577.

[jan70034-bib-0014] Dolezal, L. 2015. “The Phenomenology of Shame in the Clinical Encounter.” Medicine, Health Care, and Philosophy 18, no. 4: 567–576. 10.1007/s11019-015-9654-5. 26104831

[jan70034-bib-0015] Ferguson, T. J. , H. L. Eyre , and M. Ashbaker . 2000. “Unwanted Identities: A Key Variable in Shame—Anger Links and Gender Differences in Shame.” Sex Roles 42, no. 3/4: 133–157.

[jan70034-bib-0016] GKV . 2025. Hebammenhilfevertrag. Anlage 1.3 Vergütungsverzeichnis [Midwife Assistance Contract. Appendix 1.3 Remuneration Schedule]. https://www.gkv‐spitzenverband.de/krankenversicherung/ambulante_leistungen/hebammen_geburtshaeuser/hebammenhilfevertrag/hebammenhilfevertrag.jsp.

[jan70034-bib-0017] Hennink, M. , and B. N. Kaiser . 2022. “Sample Size for Saturation in Qualitative Research: A Systematic Review of Empirical Tests.” Social Science & Medicine 292: 114523. 10.1016/j.socscimed.2021.114523. 34785096

[jan70034-bib-0018] Keltner, D. , D. Sauter , J. L. Tracy , E. Wetchler , and A. S. Cowen . 2022. “How Emotions,Relationships, and Culture Constitute Each Other: Advances in Social Functionalist Theory.” Cognition & Emotion 36, no. 3: 388–401. 10.1080/02699931.2022.2047009. 35639090

[jan70034-bib-0019] Krausé, S. S. , C. S. Minnie , and S. K. Coetzee . 2020. “The Characteristics of Compassionate Care During Childbirth According to Midwives: A Qualitative Descriptive Inquiry.” BMC Pregnancy and Childbirth 20, no. 1: 304. 10.1186/s12884-020-03001-y. 32429908 PMC7236148

[jan70034-bib-0020] Larsson, L. 2024. Unmasking Shame, Compassion and Connection at Every Stage of Life. Friare Liv.

[jan70034-bib-0021] Lewis, M. 1995. Shame: The Exposed Self. Free Press.

[jan70034-bib-0022] Lietzmann, A. 2003. Theorie der Scham. Eine Anthropologische Perspektive auf ein Menschliches Charakterisitkum. (English: Theory of Shame. An anthropological Perspective on a Human Characteristic) Dissertation Eberhard‐Karls‐Universität Tübingen.

[jan70034-bib-0023] Lincoln, Y. S. , and E. G. Guba . 1985. Naturalistic Inquiry. Sage Publications.

[jan70034-bib-0024] Luce, A. , M. Cash , V. Hundley , H. Cheyne , E. van Teijlingen , and C. Angell . 2016. “Is It Realistic? The Portrayal of Pregnancy and Childbirth in the Media.” BMC Pregnancy and Childbirth 16: 40. 10.1186/s12884-016-0827-x. 26928660 PMC4770672

[jan70034-bib-0025] Martin, K. A. 2003. “Giving Birth Like a Girl.” Gender and Society 17, no. 1: 54–72. http://www.jstor.org/stable/3081814.

[jan70034-bib-0026] Mathias, L. A. , D. Davis , and S. Ferguson . 2021. “Salutogenic Qualities of Midwifery Care: A Best‐Fit Framework Synthesis.” Women and Birth 34, no. 3: 266–277. 10.1016/j.wombi.2020.03.006. 32327385

[jan70034-bib-0027] McHugh, M. C. 2020. “Menstrual Shame: Exploring the Role of ‘Menstrual Moaning’.” In The Palgrave Handbook of Critical Menstruation Studies, edited by C. Bobel , I. T. Winkler , B. Fahs , K. A. Hasson , E. A. Kissling , and T. A. Roberts , 409–422. Springer Nature. 10.1007/978-981-15-0614-7_32.

[jan70034-bib-0028] McKay, S. , and J. Roberts . 1990. “Obstetrics by Ear. Maternal and Caregiver Perceptions of the Meaning of Maternal Sounds During Second Stage Labor.” Journal of Nurse‐Midwifery 35, no. 5: 266–273. 2258755 10.1016/0091-2182(90)90079-k

[jan70034-bib-0029] Miller, J. B. , and I. P. Stiver . 1997. The Healing Connection: How Women Form Relationships in Therapy and in Life. Beacon Press.

[jan70034-bib-0030] Moloney, S. 2010. “How Menstrual Shame Affects Birth.” Women and Birth 23, no. 4: 153–159. 10.1016/j.wombi.2010.03.001. 20399172

[jan70034-bib-0031] Moloney, S. , and S. Gair . 2015. “Empathy and Spiritual Care in Midwifery Practice: Contributing to Women's Enhanced Birth Experiences.” Women and Birth 28: 323–328. 10.1016/j.wombi.2015.04.009. 25997730

[jan70034-bib-0032] Nathanson, D. L. 1992. Shame and Pride: Affect, Sex, and the Birth of the Self. 1st ed. Norton.

[jan70034-bib-0033] O'Brien, B. C. , I. B. Harris , T. J. Beckman , D. A. Reed , and D. A. Cook . 2014. “Standards for Reporting Qualitative Research: A Synthesis of Recommendations.” Academic Medicine 89: 1245–1251. 10.1097/ACM.0000000000000388. 24979285

[jan70034-bib-0034] Pierce, B. 1998. “The Practice of Toning in Pregnancy and Labour: Participant Experiences.” Complementary Therapies in Nursing & Midwifery 4, no. 2: 41–46. 10.1016/s1353-6117(98)80024-3. 10025286

[jan70034-bib-0035] Roberts, J. , and S. D. Benedictis . 2019. “Childbirth on Television: A Scoping Review and Recommendations for Further Research.” Feminist Media Studies 21, no. 2: 248–264. 10.1080/14680777.2019.1690025.

[jan70034-bib-0036] Roosevelt, L. , K. Danford , and R. Zielinski . 2025. “Impolite Birth: Provider Perspectives on Vocalisation During Childbirth.” Birth (Berkeley, Calif.) 0: 1–7. 10.1111/birt.12907. PMC12612353 40062445

[jan70034-bib-0037] Rosenberg, M. B. 2003. Nonviolent Communication: A Language of Life. 2nd ed. PuddleDancer.

[jan70034-bib-0038] Sedighimornani, N. 2018. “Shame and Its Features: Understanding of Shame.” European Journal of Social Sicencies Studies 3, no. 3: 75–107. 10.5281/zenodo.1453426.

[jan70034-bib-0039] Simkin, P. 2011. “Pain, Suffering, and Trauma in Labour and Prevention of Posttraumatic Stress Disorder.” Journal of Perinatal Education 20, no. 3: 166–176. 10.1891/1058-1243.20.3.166. 22654466 PMC3209770

[jan70034-bib-0040] Snow, S. , N. F. Bernardi , N. Sabet‐Kassouf , D. Moran , and A. Lehmann . 2018. “Exploring the Experience and Effects of Vocal Toning.” Journal of Music Therapy 55: 221–250. 10.1093/jmt/thy010. 29800304

[jan70034-bib-0041] Sundberg, J. 1982. “Speech, Song, and Emotions.” In Music, Mind, and Brain: The Neuropsychology of Music, edited by M. Clynes , 137–149. Springer.

[jan70034-bib-0042] Tangney, J. P. 2001. “Constructive and Destructive Aspects of Shame and Guilt.” In Constructive & Destructive Behavior: Implicaitons for Family, School, & Society, edited by A. C. Bohart and D. J. Stipek , 127–145. American Psychological Association.

[jan70034-bib-0043] Tomkins, S. S. 1987. “Shame.” In The Many Faces of Shame, edited by D. L. Nathanson , 133–161. Guilford Press.

[jan70034-bib-0047] Valente, D. , C. Magnard , A. Koutseff , et al. 2025. “Vocal Communication and Perception of Pain in Childbirth Vocalizations.” Philosophical Transactions of the Royal Society B 380: 20240009. 10.1098/rstb.2024.0009. PMC11966154 40176506

[jan70034-bib-0044] West, C. , and D. H. Zimmerman . 1987. “Doing Gender.” Gender & Society 1, no. 2: 125–151. 10.1177/0891243287001002002.

[jan70034-bib-0045] Wiseman, T. 1996. “A Concept Analysis of Empathy.” Journal of Advanced Nursing 23: 1162–1167. 8796464 10.1046/j.1365-2648.1996.12213.x

[jan70034-bib-0046] Zinsser, L. A. , A. Marek , and I. A. Stone . 2025. “Terminology of Women's Embodied Experience of Labouring and Birthing Sounds—A Qualitative Interview Study.” Midwifery 146: 104422. 10.1016/j.midw.2025.104422. 40273543

